# Supplementary material for: Complete and de novo assembly of the Leishmania braziliensis (M2904) genome
Source: Mem Inst Oswaldo Cruz. 2018 Dec 10;114:e180438. doi: 10.1590/0074-02760180438 (PMC6319030; doi:10.1590/0074-02760180438)
Supplement: Supplementary file 1 [file 1678-8060-mioc-114-e180438-s.pdf]

Complete and *de novo* assembly of the *Leishmania braziliensis* (M2904) genome

Sandra González-de la Fuente, Esther Camacho, Ramón Peiró-Pastor, Alberto Rastrojo, Fernando Carrasco-Ramiro, Begoña Aguado, Jose M Requena/+

Universidad Autónoma de Madrid, Centro de Biología Molecular Severo Ochoa, Madrid, Spain

Leishmania braziliensis is the etiological agent of American mucosal leishmaniasis, one of the most severe clinical forms of leishmaniasis. Here, we report the assembly of the *L. braziliensis* (M2904) genome into 35 continuous chromosomes. Also, the annotation of 8395 genes is provided. The public availability of this information will contribute to a better knowledge of this pathogen and help in the search for vaccines and novel drug targets aimed to control the disease caused by this *Leishmania* species.

Key words: genome assembly - next generation sequencing - Illumina short reads - PacBio long reads - chromosomes - gene annotation

Protists of the genus *Leishmania* are causative agents of a group of diseases known as leishmaniasis, which range from self-curing cutaneous lesions to mucosal disfigurations and fatal visceral outcomes.[1] According to estimates of incidence and disease burden, leishmaniasis are among the six world's most serious parasitic diseases.[2] The medical relevance of this parasite led to push the project of deciphering its genome sequence that was undertaken by an international consortium formed by the Wellcome Sanger Institute and many laboratories around the world. As a result, the *Leishmania (L.) major* genome sequence was published in 2005,[3] providing an invaluable framework for studies in very different areas of the field.[4] Soon after, as *L. major* causes in humans a relatively benign form of cutaneous leishmaniasis, representative species of the two other forms of leishmaniasis, mucosal (*Leishmania braziliensis*) and visceral (*Leishmania infantum*) were also selected for genome sequencing projects.[5] The genome sequence of another relevant species, *Leishmania donovani*, was published a few years later.[6] Nevertheless, the genome assemblies achieved for these species were of a lower quality than that obtained for the *L. major* genome; many sequence gaps remained in the chromosomal scaffolds generated. Despite the advances in high-throughput sequencing methods and the relatively small genome size of this species (around 32-Mbp, per haploid set), the abundance in repeated sequences (0.4-1 kb in length) that are scattered along the different chromosomes[7,8] is a hurdle for sequence assemblers. In fact, most of the recent genomic assemblies reported for *Leishmania* species contain a significant number of sequence gaps and uncertainties in the structure of chromosomes.

Fortunately, third-generation sequencing technologies, having the capacity of generating long sequence reads, have emerged as valuable tools for solving many of the assembly problems arising with the use of short sequence reads.[9] In particular, single molecule real time (SMRT) sequencing, developed by Pacific Biosciences (PacBio[10]), is being incorporated as a necessary methodology for generating complete chromosomal assemblies. Recently, PacBio sequencing was used to generate a new reference genome for *L. donovani*.[11] Similarly, a complete *L. infantum* genome was obtained by our group[12] through a combination of PacBio long reads and paired-end short-reads generated by Illumina technology. In the present work, following the pipeline developed for the assembly of *L. infantum*, we present a complete assembly of the *L. braziliensis* (M2904 strain) genome into 35 chromosomes, providing the corresponding gene annotation.

In this study, the M2904 strain (MHOM/BR/75/M2904) of *L. braziliensis* was used, as it is the common reference for genomic studies.[5,13] This strain was kindly provided by Dr J Moreno (WHO Collaborating Centre for Leishmaniasis, Centro Nacional de Microbiología, Instituto de Salud Carlos IIII, Madrid, Spain). Culturing conditions and the procedure for total DNA isolation have been described elsewhere.[14]

Firstly, Illumina sequencing of total DNA was carried out. Library construction and paired-end library sequencing were performed at the Centro Nacional de Análisis Genómico (CNAG-CRG, Spain; http://www.cnag.crg.eu/) using Illumina HiSeq2000 technology. A total of 51,049,711 paired-end, 2×126 bp sequence reads were generated with a mean insert size of 301 bp. PrinseqQuality (http://prinseq.sourceforge.net/) was applied to

doi: 10.1590/0074-02760180438
Financial support: Proyecto del Ministerio de Economía, Industria y Competitividad SAF2017-86965-R (co-funded with FEDER funds), and ISCIII, project RD16/0027/0008 Red de Enfermedades Tropicales, Subprograma RETICS del Plan Estatal de I+D+I 2013-2016 and co-funded with FEDER funds (grants to BA and JMR), Genomics and NGS Core Facility at the Centro de Biología Molecular Severo Ochoa (CBMSO CSIC-UAM) which is part of the CEI UAM+CSIC, and the Norwegian Sequencing Centre, a technology platform hosted by the University of Oslo and supported by the "Functional Genomics" and "Infrastructure" programs of the Research Council of Norway and the Southeastern Regional Health Authorities. Institutional grants from the Fundación Ramón Areces and Banco de Santander to the CBMSO.
SGF and EC contributed equally to this work.
+ Corresponding author: jmrequena@cbm.csic.es
Received 16 September 2018
Accepted 22 November 2018

quality filtering/trimming of reads (cut-off value of 20, quality phred score), and only reads with length ≥ 60-nt were used (Fig. 1). Finally, 50,619,063 filtered reads were assembled using the CLC Genomics Workbench version 5.0 (CLC Bio). However, the results were not satisfactory as the genome was assembled into 2,686 scaffolds with 3,888 sequence gaps remaining.

To improve the assembly, PacBio sequencing was undertaken. A total of 276,308 pre-filtered reads with a median size of 16,600 nucleotides were generated on a PacBio RS II sequencing instrument. The Norwegian Sequencing Centre (www.sequencing.uio.no) provided the sequencing service. As outlined in Fig. 1, firstly, quality trimming of PacBio reads was performed by default in the HGAP pipeline (P_filter module). *De novo* genome assembly was carried out following a hierarchical genome-assembly process (HGAP[15]), using the HGAP3 (Pacific Biosciences, SMRT Analysis Software v2.3.0) and HGAP4 (Pacific Biosciences, SMRT Link 4.0.0) tools. Three different assembly strategies were used by varying the software tool and the size of the expected genome: 34 and 35 Mbp, using HGAP3, and 35 Mbp using HGAP4; as a result, 162, 151 and 90 contigs were obtained, respectively. The HGAP4-generated contigs were selected for further analysis. Forty-four out of the 90 contigs were determined to be spurious and discarded, giving their disproportionately low coverage (mean < 40×) and/or short length (< 15-Kb). Some of these contigs corresponded to maxicircle sequences, even though a complete sequence for the *L. braziliensis* maxicircle molecule could not be generated. The rest of the discarded contigs showed a chimeric structure, being composed by *Leishmania* sequences present in two or more regions of the chromosomal-size contigs, probably due to assembly artefacts.

The remaining 46 contigs (see Fig. 1), having mean coverage of 96× (Table), were preliminary assigned to chromosomes by BLAST analysis[16] using the *L. major* (Friedlin strain) chromosomes as reference.[17] This analysis indicated that 27 of the contigs represented complete *L. braziliensis* chromosomes. The remaining 19 contigs could be ordered into continuous chromosomes without any sequence gaps (Fig. 2) using different assemblers. In particular, SSPACE-standard [version 2.0 with extension option (-x 1)] software[18] was useful to join the contigs belonging to chromosomes 8 and 9; SSPACE-LongReads (v 1-1),[19] a tool which uses only the longest PacBio subreads, was successful for joining the two contigs forming the chromosome 27. The two contigs of the chromosome 10 were merged with pyScaf (v1) software (https://github.com/lpryszcz/pyScaf),

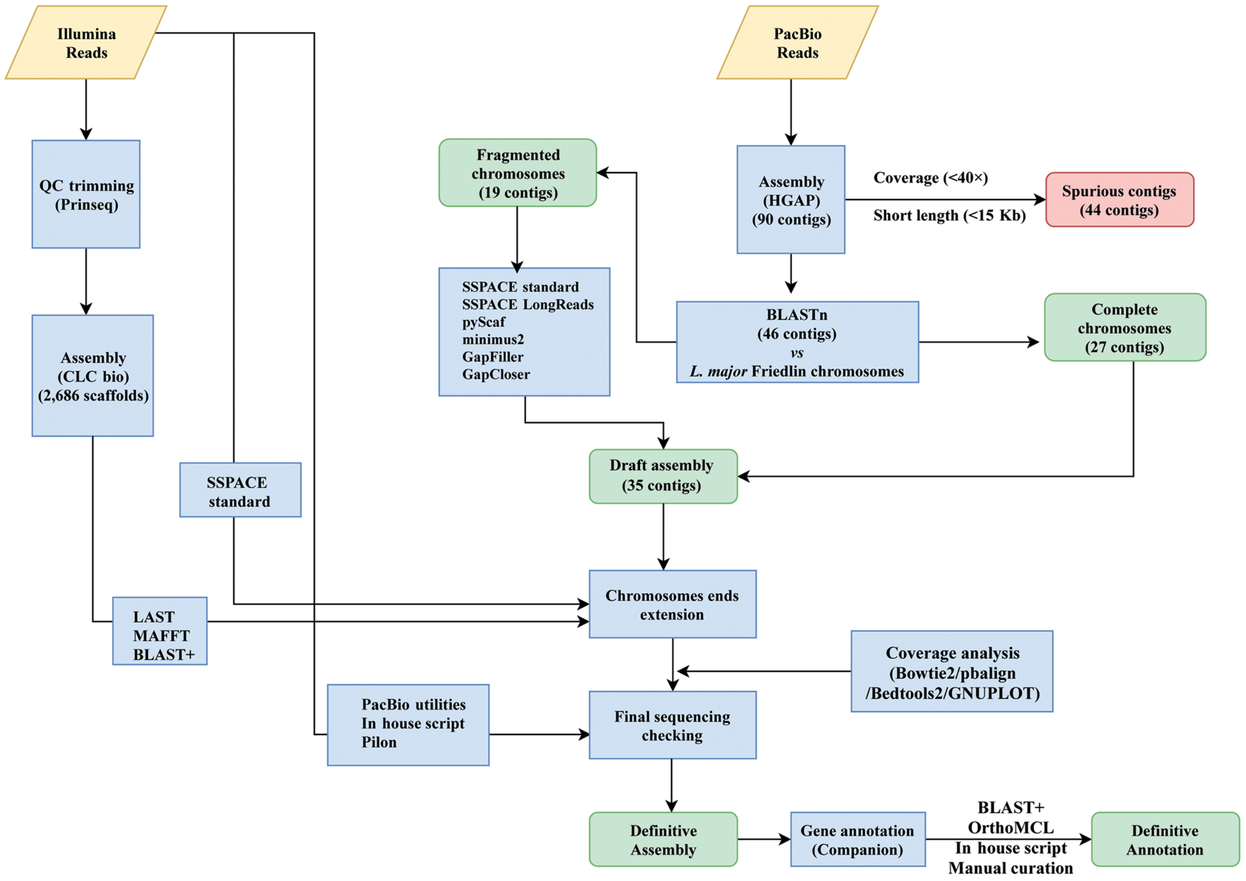

Fig. 1: schematic overview of the *Leishmania braziliensis* genome assembly workflow. Input files (Illumina and PacBio reads) are shown in yellow rhomboids. Both different assembly processes and software used are represented in blue boxes. Output files are shown in green boxes and discarded data are shown in red boxes.

TABLE

Features of the Leishmania braziliensis assembled genomes and sequencing strategies

Features	Assembly-2007[5]	Assembly-2011[28]	Assembly-2018[13]	This work
Chromosomes (scaffolds)	37	35	3782	35
Number of contigs	1041	103	13601	35
N50	57,784 bp	-	20,600 bp	1,063,631 bp
Annotated genes	8428	-	8161	8395
Annotated CDS:	8153	8357	8001	8244
- Functional annotation	-	-	-	4862
- Hypothetical proteins	-	-	-	3382
Annotated pseudogenes	161	-	65	33
Annotated structural genes	114	-	95	118
Number of gaps	2097	-	3352	0
Number of Ns	92,079	-	~316,778	0
Haploide genome size (bp)	31,996,772	31,997,773	30,009,653	32,301,632
Coverage (mean) [sequencing method]	6× [Capillary sequencing]	105× [Illumina];<br>7× [Capillary]	88× [Illumina]	363× [Illumina];<br>96× [PacBio]

-: data not found.

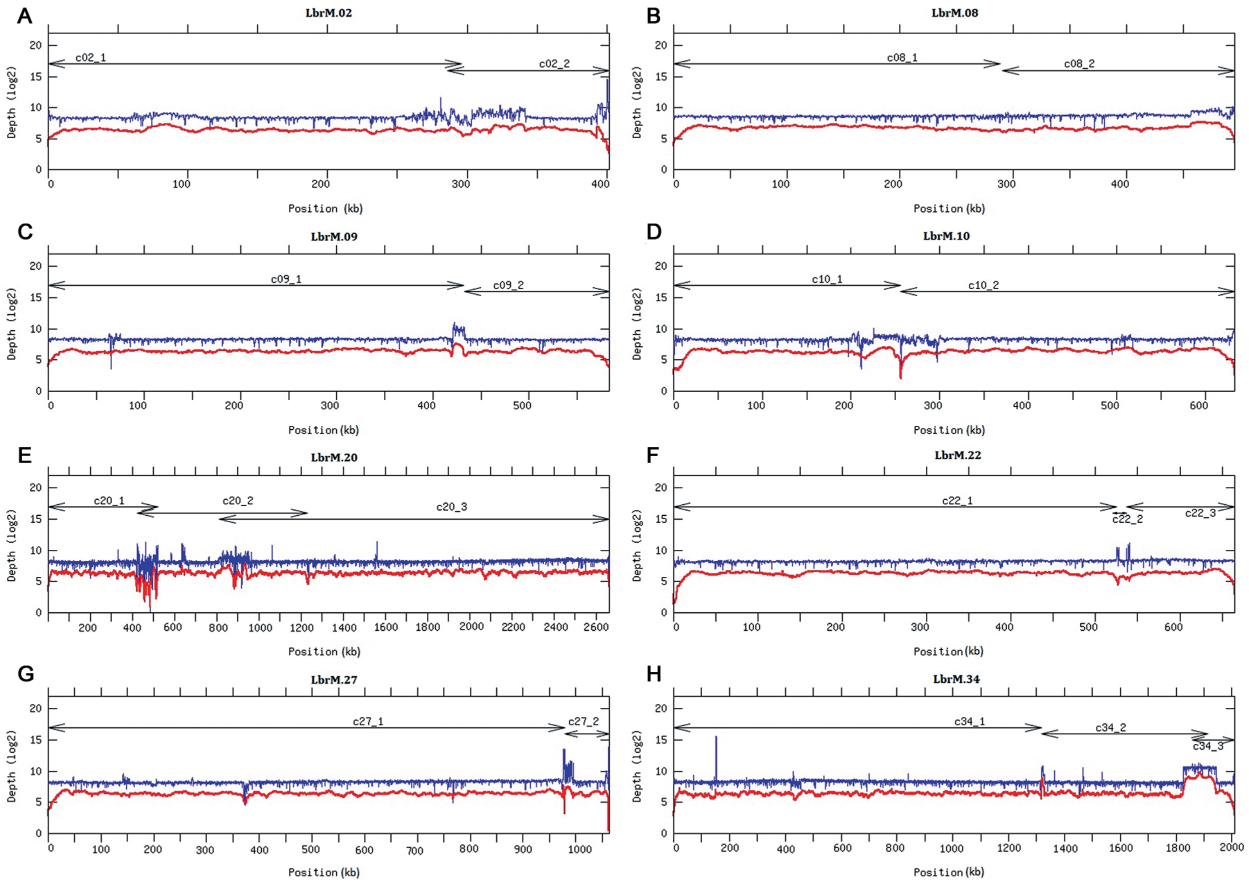

Fig. 2: read-depth analysis throughout the chromosomes formed by the union of two or more PacBio-assembled contigs. Coverage (log2 value) was appointed by sliding window analysis (bin 200 bp) with either Illumina (in blue) and PacBio (in red) reads, along chromosomes 2, 8, 9, 10, 20, 22, 27 and 34. Contigs lengths are shown by arrowheads lines. Chromosomes 2, 20, and 22 (panels A, E and F) were joined using the minimus 2 assembler. Chromosomes 8 (panel B) and 9 (panel C) were joined by the SSPACE-standard tool. Moreover, chromosome 27 (panel G) was joined by the SSPACE-LongRead tool and chromosome 10 (panel D) was joined with the pyScaf scaffolder tool. Finally, pyScaf and minimus2 were necessary for joining chromosome 34 (panel H).

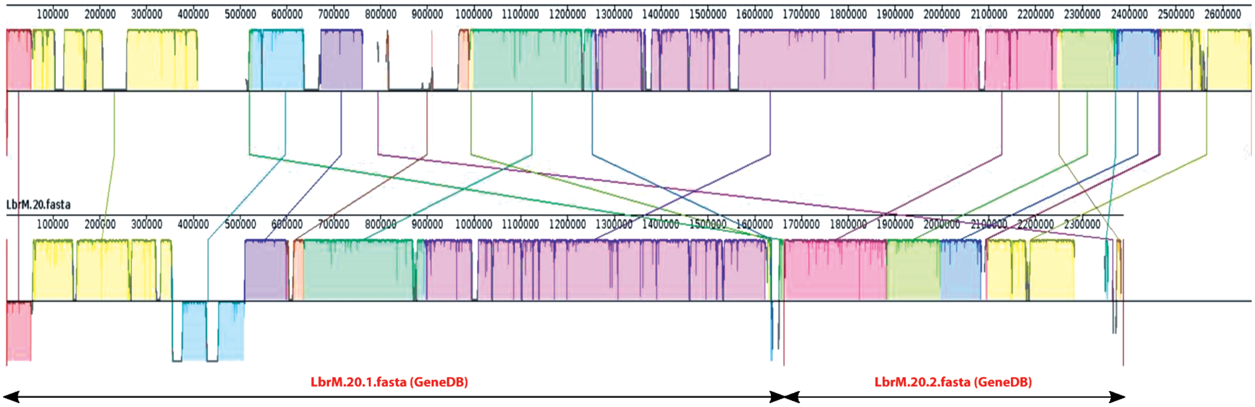

Fig. 3: schematic illustration of chromosomal fusion (20 and 34) in the chromosome 20 of *Leishmania braziliensis* genome. Pairwise alignments between the *L. braziliensis* newly assembled genome (top) and the current reference genome (bottom), which is fragmented in two scaffolds (LbrM.20.1 and LbrM.20.2), were generated using Mauve genome alignment tool with default settings. This tool uses different colors to represent synteny blocks. Sections located underneath the x-axis show inversion events. The sizes of the two contigs of the current reference genome for chromosome 20 are shown by lines with arrow-heads.

which aligns long reads onto the contigs and identifies the reads that connect them. Three contigs were identified for chromosome 34, and joined by the combination of two tools: contig1 and contig2 could be joined by pyScaf, and the union between contig 2 and contig 3 was attained by minimus2 (v2),[20] which uses NUCmer (v3.1)[21] to compute overlaps between contigs. Finally, the contigs belonging to the three remaining chromosomes (2, 20 and 22) were joined with minimus2.

Afterwards, the accuracy of the joining points was evaluated using the Illumina paired-end reads. The gap-size in each chromosome was calculated and closed by combining the results generated with the tools GapCloser (v1.12-r6)[22] and Gapfiller.[23] GapFiller (v1.10) was run with the parameters -g 3 (three allowed gaps in Bowtie alignment) and -i 100 (number of iterations to fill a gap). Additionally, the contigs generated from the Illumina sequencing reads (see above) were aligned to the *de novo* assembled chromosomes using LAST aligner (v.8, http://last.cbrc.jp/) with -uNEAR option. We found nine Illumina contigs that aligned at the ends of chromosomes 8, 15, 16, 20, 21, 22, 23 and 27, but having overhanging sequences. Extension of these chromosomes was performed using MAFFT multiple-aligner (v.7.313). To extend the rest of chromosomes, the SSPACE-standard software was used.

Coverage analysis on the final chromosomal assemblies was performed using Illumina and PacBio reads [see Supplementary data (Figs 1-35)]. Illumina reads were aligned with Bowtie2 (v.2.34.3; option: --local), and PacBio bax.h5 reads were aligned with the pbalign (BLASR, v.0.2.0) tool. Coverage analysis was performed from each alignment along the 35 chromosomes using the GenomeCoverageBed tool (v.2.25.0, using -d option, which informs the depth at each genome position) of BEDtools2. The graphical coverage plots files were generated with GNUPLOT (v.4.6, patchlevel 2; http://www.gnuplot.info/). A continuous distribution of reads was found in all chromosomes, and the reads coverage in the eight chromosomes generated by joining of two or three contigs are shown in Fig. 2. The joining points of the chromosomes assembled initially in two or more contigs correspond to regions with tandemly repeated genes (or other repeated elements), and, according to the uneven coverage observed at these points in some of the chromosomes (Fig. 2), it is likely that the exact number of repeats has still not been determined.

At this point, a detailed sequence checking was performed using PacBio-utilities (v.1; https://github.com/douglasgscofield/PacBio-utilities), designed to correct deletions and insertions introduced with some frequency, mainly in homopolymer strings, by the PacBio sequencing. Corrections were dictated by the sequence derived from Illumina reads, which have higher accuracy than the PacBio ones. Sequence insertions/deletions were corrected when they were supported by more than ten Illumina reads and the indel was present in 80% (or above) of the reads mapping the concerned position. Additionally, a second correction step was carried out using an in-house Python script, which uses the output generated by the Pilon tool (v.1.2.2, using option: --diploid).[24] As a result, 556 positions were corrected, of which 161 corresponded to sequence deletions and 395 to insertions. The accuracy of these modifications was checked by visualisation of Illumina reads mapped to the assembled genome using the IGV (v.2.3) tool.[25]

Gene annotation of both protein-coding genes and known non-coding RNAs on the new assembled *L. braziliensis* genome was performed using Companion web server, using the default settings and selecting the *L. major* (Friedlin) annotation as a reference. Additionally, OrthoMCL web version[26] and BLAST searches were used to complement the annotations generated by Companion. Afterwards, all data were combined into a GFF3 file by an in-house Python script. Finally, a manual curation by IGV visualisation of the annotated genes was performed. The annotation file is provided as a supplementary dataset. As summarised in Table, a total of 8,395 genes have been annotated, of which 8,277 correspond to protein-coding genes (including 33 pseudogenes) and 118 are genes coding for structural and/or functional RNAs (i.e., rRNAs, tRNAs and snoRNAs).

To visually illustrate the changes in the chromosomal architecture found in the assembly reported here regarding the current reference genome (GeneDB), a comparison of chromosome 20 in both assemblies is shown in Fig. 3. In the current reference, this chromosome is assembled in two separate scaffolds, whereas a continuous chromosome was assembled in this work. Moreover, several regions were assembled in a different order. The availability of a robust genome sequence, as the one presented in this work, is a valuable resource for studies addressing whole-organism aspects following either genomics, transcriptomics or proteomics approaches. Nevertheless, to date, there is not any genome assembly that can be considered as set in stone, and the *L. braziliensis* genome reported here is not an exception. In particular, we are aware that the structure of the extremities for several of the assembled chromosomes has not been determined definitively, as the telomeric hexanucleotide repeats were not assembled for all the chromosomal ends. The *Leishmania* chromosomes contain subtelomeric regions consisting of repetitive sequences with variable length and number of repeats that are often chromosome-specific, and for these reasons dedicated methodological approaches are required for accurate determination of their structure.[27]

Data availability - Genomic raw data have been deposited in The European Nucleotide Archive (ENA; http://www.ebi.ac.uk/ena/). Also, the assembled genome and annotations files were uploaded under the Study accession number PRJEB25922 and study unique name ena-STUDY-CBMSO-09-04-2018-08:02:47:223-4008. Additionally, the new *L. braziliensis* genome sequence and annotations are available at the Leish-ESP web site (http://leish-esp.cbm.uam.es/).

AUTHORS' CONTRIBUTION

FCR, BA and JMR - Conceived and designed the experiments; JMR - parasites cultures and preparation of DNA; SGF, RPP and AR - genome assembly; SGF and EC - genome annotation; SGF, EC and JMR - drawing of figures, drafted the manuscript; JMR and BA edited the final manuscript. All authors contributed to the writing, have read and approved the final manuscript.

REFERENCES

1. Herwaldt BL. Leishmaniasis. Lancet. 1999; 354(9185): 1191-9.

2. Alvar J, Velez ID, Bern C, Herrero M, Desjeux P, Cano J, et al. Leishmaniasis worldwide and global estimates of its incidence. PLoS One. 2012; 7(5): e35671.

3. Ivens AC, Peacock CS, Worthey EA, Murphy L, Aggarwal G, Berriman M, et al. The genome of the kinetoplastid parasite, *Leishmania major*. Science. 2005; 309(5733): 436-42.

4. Cantacessi C, Dantas-Torres F, Nolan MJ, Otranto D. The past, present, and future of *Leishmania* genomics and transcriptomics. Trends Parasitol. 2015; 31(3): 100-8.

5. Peacock CS, Seeger K, Harris D, Murphy L, Ruiz JC, Quail MA, et al. Comparative genomic analysis of three *Leishmania* species that cause diverse human disease. Nat Genet. 2007; 39(7): 839-47.

6. Downing T, Imamura H, Decuypere S, Clark TG, Coombs GH, Cotton JA, et al. Whole genome sequencing of multiple *Leishmania donovani* clinical isolates provides insights into population structure and mechanisms of drug resistance. Genome Res. 2011; 21(12): 2143-56.

7. Ubeda JM, Raymond F, Mukherjee A, Plourde M, Gingras H, Roy G, et al. Genome-wide stochastic adaptive DNA amplification at direct and inverted DNA repeats in the parasite *Leishmania*. PLoS Biol. 2014; 12(5): e1001868.

8. Requena JM, Rastrojo A, Garde E, Lopez MC, Thomas MC, Aguado B. Genomic cartography and proposal of nomenclature for the repeated, interspersed elements of the *Leishmania major* SIDER2 family and identification of SIDER2-containing transcripts. Mol Biochem Parasitol. 2017; 212: 9-15.

9. Goodwin S, McPherson JD, McCombie WR. Coming of age: ten years of next-generation sequencing technologies. Nat Rev Genet. 2016; 17(6): 333-51.

10. Eid J, Fehr A, Gray J, Luong K, Lyle J, Otto G, et al. Real-time DNA sequencing from single polymerase molecules. Science. 2009; 323(5910): 133-8.

11. Dumetz F, Imamura H, Sanders M, Seblova V, Myskova J, Pescher P, et al. Modulation of aneuploidy in *Leishmania donovani* during adaptation to different *in vitro* and *in vivo* environments and its impact on gene expression. MBio. 2017; 8(3): e00599-17.

12. González-de la Fuente S, Peiro-Pastor R, Rastrojo A, Moreno J, Carrasco-Ramiro F, Requena JM, et al. Resequencing of the *Leishmania infantum* (strain JPCM5) genome and de novo assembly into 36 contigs. Sci Rep. 2017; 7(1): 18050.

13. Coughlan S, Taylor AS, Feane E, Sanders M, Schonian G, Cotton JA, et al. *Leishmania naiffi* and *Leishmania guyanensis* reference genomes highlight genome structure and gene evolution in the Viannia subgenus. R Soc Open Sci. 2018; 5(4): 172212.

14. Ramírez CA, Requena JM, Puerta CJ. Identification of the HSP70-II gene in *Leishmania braziliensis* HSP70 locus: genomic organization and UTRs characterization. Parasit Vectors. 2011; 4: 166.

15. Chin CS, Alexander DH, Marks P, Klammer AA, Drake J, Heiner C, et al. Nonhybrid, finished microbial genome assemblies from long-read SMRT sequencing data. Nat Methods. 2013; 10(6): 563-9.

16. Camacho C, Coulouris G, Avagyan V, Ma N, Papadopoulos J, Bealer K, et al. BLAST+: architecture and applications. BMC Bioinformatics. 2009; 10: 421.

17. Alonso G, Rastrojo A, López-Pérez S, Requena JM, Aguado B. Resequencing and assembly of seven complex loci to improve the *Leishmania major* (Friedlin strain) reference genome. Parasit Vectors. 2016; 9(1): 74.

18. Boetzer M, Henkel CV, Jansen HJ, Butler D, Pirovano W, et al. Scaffolding pre-assembled contigs using SSPACE. Bioinformatics. 2011; 27(4): 578-9.

19. Boetzer M, Pirovano W. SSPACE-LongRead: scaffolding bacterial draft genomes using long read sequence information. BMC Bioinformatics. 2014; 15: 211.

20. Sommer DD, Delcher AL, Salzberg SL, Pop M. Minimus: a fast, lightweight genome assembler. BMC Bioinformatics. 2007; 8: 64.

21. Delcher AL, Phillippy A, Carlton J, Salzberg SL. Fast algorithms for large-scale genome alignment and comparison. Nucleic Acids Res. 2002; 30(11): 2478-83.

22. Luo R, Liu B, Xie Y, Li Z, Huang W, Yuan J. SOAPdenovo2: an empirically improved memory-efficient short-read de novo assembler. Gigascience. 2012; 1(1): 18.

23. Nadalin F, Vezzi F, Policriti A, GapFiller: a de novo assembly approach to fill the gap within paired reads. BMC Bioinformatics. 2012; 13(Suppl. 14): S8.

24. Walker BJ, Abeel T, Shea T, Priest M, Abouelliel A, Sakthikumar S, et al. Pilon: an integrated tool for comprehensive microbial variant detection and genome assembly improvement. PLoS One. 2014; 9(11): e112963.

25. Thorvaldsdottir H, Robinson JT, Mesirov JP. Integrative Genomics Viewer (IGV): high-performance genomics data visualization and exploration. Brief Bioinform. 2013; 14(2): 178-92.

26. Li L, Stoeckert Jr CJ, Roos DS. OrthoMCL: identification of ortholog groups for eukaryotic genomes. Genome Res. 2003; 13(9): 2178-89.

27. Chiurillo MA, Ramírez JL. Charaterization of *Leishmania major* Friedlin telomeric terminus. Mem Inst Oswaldo Cruz. 2002; 97(3): 343-6.

28. Rogers MB, Hilley JD, Dickens NJ, Wilkes J, Bates PA, Depledge DP, et al. Chromosome and gene copy number variation allow major structural change between species and strains of *Leishmania*. Genome Res. 2011; 21(12): 2129-42.
